# Supplementary material for: Predicting the effects of reservoir water level management on the reproductive output of a riparian songbird
Source: PLoS One. 2021 Feb 22;16(2):e0247318. doi: 10.1371/journal.pone.0247318 (PMC7899321; doi:10.1371/journal.pone.0247318)
Supplement: S1 Table — Productivity estimates are presented using the baseline parameters (see Table 1 for source of parameter estimate) and after individual parameters are modified by fixed amounts. Scenario 1 = long term conditions (ie the reservoir water levels observed between 1968 and 2017; Scenario 2 = early fill years (when water levels reach 435m a.s.l. by June 12); Scenario 3 = late fill years (when water levels do not reach 435m a.s.l. until after June 12); Scenario 4 = zero flooding (where reservoir water levels never exceed 435 m a.s.l). (DOCX) [file pone.0247318.s001.docx]

Table S1. Model estimated productivity ± SD of female yellow warblers (average number of fledglings per female and average number of independent young per female) over 50 breeding seasons under four reservoir water level management scenarios. Productivity estimates are presented using the baseline parameters (see Table 1 for source of parameter estimate) and after individual parameters are modified by fixed amounts. Scenario 1 = long term conditions (ie the reservoir water levels observed between 1968 and 2017; Scenario 2 = early fill years (when water levels reach 435m a.s.l. by June 12); Scenario 3 = late fill years (when water levels do not reach 435m a.s.l. until after June 12); Scenario 4 = zero flooding (where reservoir water levels never exceed 435 m a.s.l).

| Parameter | Model variant | Parameter value(s) | Scenario | Fledglings | Independent young |
| --- | --- | --- | --- | --- | --- |
| Parasitism rate | Model | 0.19 | 1 | 2.25 ± 0.39 | 1.37 ± 0.51 |
|  | Model | 0.19 | 2 | 2.06 ± 0.43 | 1.06 ± 0.51 |
|  | Model | 0.19 | 3 | 2.21 ± 0.39 | 1.44 ± 0.40 |
|  | Model | 0.19 | 4 | 2.28 ± 0.37 | 1.68 ± 0.27 |
|  | Model - 50% | 0.095 | 1 | 2.26 ± 0.41 | 1.39 ± 0.47 |
|  | Model - 50% | 0.095 | 2 | 2.09 ± 0.38 | 1.04 ± 0.44 |
|  | Model - 50% | 0.095 | 3 | 2.27 ± 0.43 | 1.53 ± 0.45 |
|  | Model - 50% | 0.095 | 4 | 2.41 ± 0.40 | 1.74 ± 0.34 |
|  | Model +50% | 0.285 | 1 | 2.09 ± 0.35 | 1.35 ± 0.41 |
|  | Model +50% | 0.285 | 2 | 2.03 ± 0.44 | 0.99 ± 0.40 |
|  | Model +50% | 0.285 | 3 | 2.16 ± 0.37 | 1.42 ± 0.38 |
|  | Model +50% | 0.285 | 4 | 2.21 ± 0.39 | 1.63 ± 0.31 |
| Abandonment rate | Model | 0.19 | 1 | 2.25 ± 0.39 | 1.37 ± 0.51 |
|  | Model | 0.19 | 2 | 2.06 ± 0.43 | 1.06 ± 0.51 |
|  | Model | 0.19 | 3 | 2.21 ± 0.39 | 1.44 ± 0.40 |
|  | Model | 0.19 | 4 | 2.28 ± 0.37 | 1.68 ± 0.27 |
|  | Model - 50% | 0.095 | 1 | 2.11 ± 0.41 | 1.44 ± 0.50 |
|  | Model - 50% | 0.095 | 2 | 2.05 ± 0.40 | 0.99 ± 0.43 |
|  | Model - 50% | 0.095 | 3 | 2.21 ± 0.36 | 1.41 ± 0.38 |
|  | Model - 50% | 0.095 | 4 | 2.28 ± 0.38 | 1.66 ± 0.29 |
|  | Model +50% | 0.285 | 1 | 2.11 ± 0.45 | 1.21 ± 0.51 |
|  | Model +50% | 0.285 | 2 | 2.04 ± 0.36 | 0.98 ± 0.43 |
|  | Model +50% | 0.285 | 3 | 2.21 ± 0.42 | 1.50 ± 0.38 |
|  | Model +50% | 0.285 | 4 | 2.29 ± 0.37 | 1.67 ± 0.29 |
| Daily Nest Survival Rate | Model | 0.9712, 0.9577 | 1 | 2.25 ± 0.39 | 1.37 ± 0.51 |
| (eggs, nestlings) | Model | 0.9712, 0.9577 | 2 | 2.06 ± 0.43 | 1.06 ± 0.51 |
|  | Model | 0.9712, 0.9577 | 3 | 2.21 ± 0.39 | 1.44 ± 0.40 |
|  | Model | 0.9712, 0.9577 | 4 | 2.28 ± 0.37 | 1.68 ± 0.27 |
|  | Model - 1% | 0.9615, 0.9481 | 1 | 1.85 ± 0.51 | 1.17 ± 0.51 |
|  | Model - 1% | 0.9615, 0.9481 | 2 | 1.64 ± 0.42 | 0.74 ± 0.38 |
|  | Model - 1% | 0.9615, 0.9481 | 3 | 1.88 ± 0.34 | 1.24 ± 0.33 |
|  | Model - 1% | 0.9615, 0.9481 | 4 | 1.97 ± 0.36 | 1.45 ± 0.29 |
|  | Model + 1% | 0.9809, 0.9673 | 1 | 2.46 ± 0.48 | 1.55 ± 0.56 |
|  | Model + 1% | 0.9809, 0.9673 | 2 | 2.34 ± 0.44 | 1.07 ± 0.54 |
|  | Model + 1% | 0.9809, 0.9673 | 3 | 2.57 ± 0.35 | 1.75 ± 0.36 |
|  | Model + 1% | 0.9809, 0.9673 | 4 | 2.65 ± 0.32 | 1.94 ± 0.27 |
| Juvenile Survival | Model | 0.214, 0.729 | 1 | 2.25 ± 0.39 | 1.37 ± 0.51 |
| (flooded, not flooded) | Model | 0.214, 0.729 | 2 | 2.06 ± 0.43 | 1.06 ± 0.51 |
|  | Model | 0.214, 0.729 | 3 | 2.21 ± 0.39 | 1.44 ± 0.40 |
|  | Model | 0.214, 0.729 | 4 | 2.28 ± 0.37 | 1.68 ± 0.27 |
|  | Model + 100% | 0.428, 0.729 | 1 | 2.25 ± 0.39 | 1.48 ± 0.43 |
|  | Model + 100% | 0.428, 0.729 | 2 | 2.06 ± 0.44 | 1.26 ± 0.43 |
|  | Model + 100% | 0.428, 0.729 | 3 | 2.21 ± 0.39 | 1.52 ± 0.34 |
|  | Model + 100% | 0.428, 0.729 | 4 | 2.28 ± 0.37 | 1.68 ± 0.27 |
|  | Model - equal | 0.729, 0.729 | 1 | 2.25 ± 0.39 | 1.64 ± 0.31 |
|  | Model - equal | 0.729, 0.729 | 2 | 2.06 ± 0.44 | 1.51 ± 0.34 |
|  | Model - equal | 0.729, 0.729 | 3 | 2.21 ± 0.39 | 1.63 ± 0.28 |
|  | Model - equal | 0.729, 0.729 | 4 | 2.28 ± 0.37 | 1.68 ± 0.27 |
