# Supplementary material for: Predicting the effects of reservoir water level management on the reproductive output of a riparian songbird
Source: PLoS One. 2021 Feb 22;16(2):e0247318. doi: 10.1371/journal.pone.0247318 (PMC7899321; doi:10.1371/journal.pone.0247318)
Supplement: S1 Data — (DOCX) [file pone.0247318.s002.docx]

**Matlab files**

File 1. Global variables.

File 2. Model Parameters

File 3. Renesting model

The following files run a stochastic model that calculates the average number of eggs, nestlings, fledglings and independent young produced by a female songbird in riparian habitat impacted by reservoir operations. The model uses data and parameters from a long-term study of Yellow Warblers in riparian habitat within the footprint of Arrow Lakes Reservoir near Revelstoke, BC, Canada.

The code was written by Eirikur Palsson, Dept. of Biological Sciences, SFU

The text would need to be copied and saved as .m files in order to be run using Matlab

**File 1. Global variables (save as global_var.m)**

global cNUM nrNests suNests avNests avsuNests avprNests proFE

global WL WLT DAY RPTS NRF PAR PARa PFSwet PFSdry RNS DNSe DNSn FEsuc

global meNE sdNE meNH sdNH NE NH CS RPTnr

global DFE EGGS EGGSt DOF

global cFLE FLE FLEt NSTL NSTLt IND INDt

global DOA cDOA FLD

global avEGGS avNSTL avFLE avIND g_aveEGS YEAR nrYEARS EF

global wetness % 1=all years 2=early, 3=late, 4=dry

global dirname

**File 2. Model parameters (save as ywar_ibm_parameters.m)**

function ywar_ibm_parameters

run global_var.m

nrYEARS = [50 22 28 1]; % number of years with reservoir water level data for each scenario

RPTS = 50; % number of years simulated

NRF= 35; % number and identity of female

%Fixed parameters

NE = zeros(NRF,1,'single'); % Nesting Elevation

NH = zeros(NRF,1,'single'); % Nesting Height

meNE = 438.5; % mean Nesting Elevation

sdNE = 0.9; % standard deviation Nesting Elevation

meNH = 2.1; % mean Nesting Height

sdNH = 1.1; % standard deviation Nesting Height

cNUM = 0; % Clutch number at start of season

CS = zeros(NRF,1,'int16'); % Clutch Size, (CS=9.603-0.033*DOY)

PAR = 0.19; % 0.19 = Probability of parasitism

PARa = 0.19; % 0.19 Probability of abandonment after parasitism

DNSe = 0.9712; % Nest with eggs daily survival probability

DNSn = 0.9577; % Nest with nestlings daily survival probability

EF = 0.818; % Probability of egg failure or nestling death

PFSdry= 0.729; % survival of fledglings no water on ground

PFSwet =0.214 ; % survival of fledglings water on ground

DFE = zeros(NRF,5,'int16'); % 1st 2nd and 3rd clutch initiation day mean±sd = 0±7

EGGS = zeros(NRF,5,'single'); % eggs laid for 1st 2nd 3rd clutch

EGGSt = zeros(NRF,1,'single'); % total eggs laid by female per season

NSTL =zeros(NRF,5, 'single'); % number of nestlings in 1st 2nd and 3rd brood

NSTLt =zeros(NRF,1, 'single'); % number of nestlings in 1st 2nd and 3rd brood

FLE = zeros(NRF,5,'single'); % Number of Fledglings 1st 2nd 3rd clutch

FLEt = zeros(NRF,1,'single'); % total fledglings produced by female per season

IND = zeros(NRF,5,'single'); % Number of Independent Young 1st 2nd 3rd clutch

INDt = zeros(NRF,1,'single'); % total of Independent Young that survive per female

nrNests = zeros(NRF,1,'int16'); % number of Nests initiated per female

suNests = zeros(NRF,1,'int16'); % number of successful per female

FEsuc = zeros(NRF,1,'int16'); % females that produce at least one fledgling, 0 or 1

cDOA = zeros(NRF,1,'int16'); % current date of abandonment, if 0 then no abandonment

DOA = zeros(NRF,5,'int16'); % date of abandonment 1st 2nd 3rd; = 0 is no abandonment

DOF = zeros(NRF,5,'int16'); % date of flooding for 1st 2nd 3rd clutch, if 0 then no flooding

FLD = zeros(NRF,5,'int16'); % fledging date 1st 2nd 3rd clutch

RNS = zeros(NRF,1,'int16'); % renesting flag when 1 then bird renests

% Parameter for total number after repeats.

avEGGS = zeros(RPTS,1,'single'); % average number of eggs laid in each repeat

avIND = zeros(RPTS,1,'single'); % average number of Independent Young in each repeat

avFLE = zeros(RPTS,1,'single'); % average number of Fledglings in each repeat

avNests = zeros(RPTS,1,'single'); % average number of Nests initiated per year

avsuNests = zeros(RPTS,1,'single'); % average number of Nests that survive per year

avprNests = zeros(RPTS,1,'single'); % proportion of Nests that survive

proFE = zeros(RPTS,1,'single'); % proportion of females that produce at least one fledgling

g_aveEGGS=0; % average number of eggs laid for all repeats

end

**File 3.** Renesting model (save as ywar_ibm_renesting_model.m)

function ywar_ibm_renesting_model

run global_var.m

ywar_ibm_parameters

wetness=1; % options 1=all years 2=early, 3=late, 4= dry(max is 435m)

filename='Reservoir_levels_by_Year.xlsx';

RPTS % number of years simulated

% ------ Read in daily Water levels for years with specified Management Scenario -----

WLT = readtable(filename,'ReadVariableNames',true,'Sheet',wetness);

WL = table2array(WLT(:,2:(1+nrYEARS(wetness)) ));

% WL = zeros(366,50,'single');

% WL[DAY, YEAR]=water levels on given day from "Reservoir levels by Year.xlsx"

% ------ End: Read in daily Water levels -------

%--------- Create directory for specific management scenario = wetness 1 2 3 4

dirname = sprintf('wetness%d',wetness);

mkdir(dirname)

fileID = fopen('Averages.txt','w');

%Averages.txt lists the averages of the various variables (eggs, nestl, fledl,inde) for each repeat.

%At the end of the Averages.txt file the average of all the repeats is listed.

rng(13) % seed for random generator use same seed to directly compare each

% ------------- Run all the repeats for the specified Management Scenario -----------------------

for tr = 1:RPTS % for years 1- RPTS=50)

RPTnr = tr;

DAY=1; % First day

cNUM = 1; % Clutch number

EGGSt = zeros(NRF,1,'single'); % total eggs laid by female per season

NSTLt = zeros(NRF,1,'single'); % total of nestlings that survive per female

FLEt = zeros(NRF,1,'single'); % total of nestlings that survive per female

INDt = zeros(NRF,1,'single'); % total of fledglings that survive per female

cDOA = zeros(NRF,1,'int16'); % current date of abandonment, if 0 no abandonment

DOA = zeros(NRF,5,'int16'); % date of abandonment 1st 2nd 3rd; if 0 no abandonment

RNS = zeros(NRF,1,'int16'); % renesting flag when 1 then bird renests

find_nr_indyoungproductivity(tr); % runs for each year 1-50

fprintf(fileID,'Average nr of: eggs per female = %f, fledglings= %f, indyoung = %f\n', mean(EGGSt),mean(FLEt),mean(INDt));

end % for tr = 1:RPTS

%---***********************

fprintf('Average nr of eggs per female = %f\n',mean(avEGGS));

fprintf('Average nr of nestlings per female = %f\n',mean(avNSTL));

%fprintf('Average nr of fledglings per female = %f\n',mean(avFLE));

fprintf('Average nr of indyoung per female = %f\n',mean(avIND));

fprintf('Average nr of nests made per female = %f\n',mean(avNests));

fprintf('Average nr of nests surviving per female = %f\n',mean(avsuNests));

fprintf('Proportion of nests surviving = %f\n',mean(avprNests));

fprintf('Proportion of females with at least one nestling surviving = %f\n',mean(proFE));

fprintf(fileID,'-------------------------------------\n');

fprintf(fileID,'Average nr of eggs per female = %f\n',mean(avEGGS));

%fprintf(fileID,'Average nr of nestlings per female = %f\n',mean(avNSTL));

fprintf(fileID,'Average nr of fledglings per female = %f\n',mean(avFLE));

fprintf(fileID,'Average nr of indyoung per female = %f\n',mean(avIND));

fprintf(fileID,'Average nr of nests made per female = %f\n',mean(avNests));

fprintf(fileID,'Average nr of nests surviving per female = %f\n',mean(avsuNests));

fprintf(fileID,'Proportion of nests surviving = %f\n',mean(avprNests));

fprintf(fileID,'Proportion of females with at least one nestling surviving = %f\n',mean(proFE));

%---***********************

fclose(fileID);

movefile('Averages.txt',dirname);

end % function ywar_ibm_renesting_model(action)

function find_nr_indyoungproductivity(tr)

% ------------ Set daily water levels and breeding phenology for the year ----------------------

run global_var.m

YEAR=randi([1 nrYEARS(wetness)]);

mDFE=158 +2.25*randn;

% -------------- Reset to zero all parameters calculated for each female

EGGS = zeros(NRF,5,'single'); % eggs laid for 1st 2nd 3rd clutch)

DOF = zeros(NRF,5,'int16'); % date of flooding for 1st 2nd 3rd clutch . if 0 then no flooding

NSTL = zeros(NRF,5,'single'); % Number of nestlings 1st 2nd 3rd clutch

FLE = zeros(NRF,5,'single'); % number of fledglings 1st 2nd 3rd clutch

IND = zeros(NRF,5,'single'); % number of ind young 1st 2nd 3rd clutch

DOA = zeros(NRF,5,'int16'); % date of abandonment/nest failure of 1st 2nd 3rd; 0 is no abandonment/ nest failure

FLD = zeros(NRF,5,'int16'); % fledging date 1st 2nd 3rd clutch

nrNests = zeros(NRF,1,'int16'); % number of nests initiated per female

suNests = zeros(NRF,1,'int16'); % number of successful nests per female

FEsuc = zeros(NRF,1,'int16'); % Female produces at least one fledgling; 0 or 1

% ----- Define nest location, start breeding season and CS of first nest for each female ------

NE = meNE + sdNE*slicesample(1,NRF,'pdf',@normpdf,'thin',2,'burnin',200); % Nesting Elev

NH = meNH + sdNH*slicesample(1,NRF,'pdf',@normpdf,'thin',2,'burnin',200); % Nesting Height

NH = max(0,NH);

DFE(:,1) = round(mDFE + 7*slicesample(1,NRF,'pdf',@normpdf,'thin',2,'burnin',200));

CS = round(9.603 - 0.033*DFE(:,1));

for i = 1:NRF

DAY = 1;

cNUM = 1; % Clutch number

EGGS(i,cNUM) = CS(i); % eggs in clutch

EGGSt(i) = EGGS(i,cNUM);

NSTL(i,cNUM) = EGGS(i,cNUM); % hatching failure is dealt with later

nrNests(i) = nrNests(i)+1; % nr of nests that are made by each female

% -------------- Parasitism and abandonment

if (rand < PAR) % Nest is parasitizsed

NSTL(i,cNUM) = NSTL(i,cNUM)-1;

if (rand < PARa) % Nest is abandoned

NSTL(i,cNUM) = 0; % all eggs die, fledge 0

cDOA(i) = ceil(DFE(i,cNUM) + CS(i)/2); % Female abandons eggs in middle of laying

DOA(i,cNUM) = cDOA(i);

DAY = DOA(i,cNUM);

end

end % if (rand < PAR)

% -------------- Hatching failure and partial brood loss

nreggs=NSTL(i,cNUM);

for ii = 1:nreggs % eggs may fail to hatch and nestlings may die

if rand>EF

NSTL(i,cNUM) = NSTL(i,cNUM)-1;

end

end

% -------------- Flooding and nest failure

if ( cDOA(i) == 0 ) % Nest is not abandoned

last_egg = DFE(i,cNUM) + CS(i) +8; % nest has eggs for this period

DAY = DFE(i,cNUM);

while (DAY <= last_egg)

if (WL(DAY,YEAR) > NE(i) + NH(i)) % nest with egg floods

DOF(i,cNUM) = DAY;

end

if ( (WL(DAY,YEAR) > NE(i) + NH(i)) || ( rand > DNSe )) % nest with eggs fails

cDOA(i) = DAY;

DOA(i,cNUM) = cDOA(i);

NSTL(i,cNUM) = 0; % all eggs die, Nestl 0

break

end

DAY = DAY + 1;

end

end % if ( cDOA(i) == 0 )

FLE(i,cNUM) = NSTL(i,cNUM);

if ( cDOA(i) == 0 ) % nest has nestlings

last_fle = DAY + 9;

while (DAY < last_fle)

if (WL(DAY,YEAR) > NE(i) + NH(i)) % nest with nestlings floods

DOF(i,cNUM) = DAY;

end

if ( (WL(DAY,YEAR) > NE(i) + NH(i)) || ( rand > DNSn )) % nest with nestlings fails

cDOA(i) = DAY;

DOA(i,cNUM) = cDOA(i);

FLE(i,cNUM) = 0; % nestlings die, fledge 0

break

end

DAY = DAY + 1;

end

end

% -------------- Post-fledging Survival

IND(i,cNUM) = FLE(i,cNUM);

if ( cDOA(i) == 0 )

suNests(i)=suNests(i)+1;

nrIND = FLE(i,cNUM);

FLD(i,cNUM) = DAY;

for j=1:nrIND

if ( WL(DAY,YEAR) > NE(i) )

if (rand > PFSwet)

IND(i,cNUM) = IND(i,cNUM) -1;

end

else

if (rand > PFSdry)

IND(i,cNUM) = IND(i,cNUM) -1;

end

end

end % for j=1:nrIND

NSTLt(i) = NSTLt(i) + NSTL(i,cNUM); % total number of nestlings for female i

FLEt(i) = FLEt(i) + FLE(i,cNUM); % total number of fledglings for female i

INDt(i) = INDt(i) + IND(i,cNUM); % total number of ind young for female i

end % if cDOA(i) == 0 )

% -------------- Determine if female re-nests after success or failure

CUTOFF = 185 ; % 192 – 7 last date nest ever initiated – nest interval

if (suNests(i)>=1)

CUTOFF = 180 ; % 187 – 7 last date nest initiated after success – nest interval

end

if (nrNests(i)>2)

CUTOFF = 176 ; % 183 - 7

end

while ( (DAY < CUTOFF) && (suNests(i) < 3) && (nrNests(i) < 4) )

% if (cNUM>=3)

% qws=23;

% end

if ( cDOA(i) == 0 ) % determine if re-nests after success

LOGIT = 34.3236-0.2108*single(DAY);

if (rand < exp(LOGIT)/(1+ exp(LOGIT)) )

RNS(i)=1;

DFE(i,cNUM+1) = DAY + 7;

nrNests(i) = nrNests(i)+1; % a new nest is made

else

DAY=CUTOFF+1;

end

else

LOGIT = 35.534-0.208*single(DAY); % determine if re-nests after failure

if (rand < exp(LOGIT)/(1+ exp(LOGIT)) );

RNS(i)=1;

DFE(i,cNUM+1) = DOA(i,cNUM) +6;

cDOA(i) = 0;

nrNests(i) = nrNests(i)+1; % a new nest is made

else

DAY=CUTOFF+1;

end

end % if ( cDOA(i) == 0 )

if (RNS(i) == 1) % renests so set nest elevation and height

NE(i) = meNE + sdNE*randn;

NH(i) = max(0,meNH + sdNH*randn);

cNUM = cNUM +1;

CS(i) = round(9.603 - 0.033*single(DFE(i,cNUM)));

RNS(i) = 0;

EGGS(i,cNUM) = CS(i); % eggs in clutch

EGGSt(i) = EGGSt(i) + EGGS(i,cNUM); % add to Total eggs laid

NSTL (i,cNUM) = EGGS(i,cNUM);

renest(i,DFE(i,cNUM)); % go to function renest(i,cNUM,DFEx)

end

end % while (DAY < CUTOFF )

if (suNests(i) >= 1)

FEsuc(i)=1;

end

end % for i = 1:NRF

%----------------- Create summary table with headers

FEnr=[1:NRF]'; %' Number of females in table

DAarray = table(FEnr,EGGS(:,1),EGGS(:,2),EGGS(:,3),EGGSt,NSTLt,FLEt,INDt,DFE(:,1),...

FLD(:,1),FLD(:,2),FLD(:,3),nrNests,suNests,FEsuc(:,1),DOA(:,1),DOA(:,2),DOA(:,3),DOF(:,1),DOF(:,2),DOF(:,3));

DAarray.Properties.VariableNames = {'Female','EGG1','EGG2','EGG3','EGGS','Nestl',...

'Flegdl','Indep','DFE','FLD1','FLD2','FLD3','nrNes','suNes','suFE','DOA1','DOA2','DOA3','DOF1','DOF2','DOF3'};

avEGGS(tr) = mean(EGGSt);

avNSTL(tr) = mean(NSTLt);

avFLE(tr) = mean(FLEt);

avIND(tr) = mean(INDt);

avNests(tr) = mean(nrNests);

avsuNests(tr) = mean(suNests);

avprNests(tr) = avsuNests(tr)/avNests(tr);

proFE(tr) = mean(FEsuc);

dataname=sprintf('BirdDataR%d.xlsx',RPTnr);

writetable(DAarray,dataname,'Sheet',1);

movefile(dataname,dirname);

end % end function Findfind_nr_indyoungproductivity

function renest(i,DFEx) % run through re-nesting loop

run global_var.m

% -------------- Parasitism and abandonment

if (rand < PAR) % Nest is parasitizsed

NSTL(i,cNUM) = NSTL(i,cNUM)-1;

if (rand < PARa) % Nest is abandoned

NSTL(i,cNUM) = 0; % all eggs die, fledge 0

cDOA(i) = DFEx + CS(i)/2 + 1; % female abandons nest in middle of laying

DOA(i,cNUM) = cDOA(i);

DAY = DOA(i,cNUM);

end

end % if (rand < PAR)

% -------------- Hatching failure and partial brood loss

nreggs=NSTL(i,cNUM);

for ii = 1:nreggs % eggs may fail to hatch and nestlings may die

if rand>EF

NSTL(i,cNUM) = NSTL(i,cNUM)-1;

end

end

% -------------- Flooding and nest failure

if ( cDOA(i) == 0 ) % Nest is not abandoned

last_egg = DFE(i,cNUM) + CS(i) +8; % nest has eggs for this period

DAY = DFE(i,cNUM);

while (DAY <= last_egg)

if (WL(DAY,YEAR) > NE(i) + NH(i)) % nest with egg floods

DOF(i,cNUM) = DAY;

end

if ( (WL(DAY,YEAR) > NE(i) + NH(i)) || ( rand > DNSe )) % nest with eggs fails

cDOA(i) = DAY;

DOA(i,cNUM) = cDOA(i);

NSTL(i,cNUM) = 0; % all eggs die, Nestl 0

break

end

DAY = DAY + 1;

end

end % if ( cDOA(i) == 0 )

FLE(i,cNUM) = NSTL(i,cNUM);

if ( cDOA(i) == 0 ) % nest has nestlings

last_fle = DAY + 9;

while (DAY < last_fle)

if (WL(DAY,YEAR) > NE(i) + NH(i)) % nest with nestlings floods

DOF(i,cNUM) = DAY;

end

if ( (WL(DAY,YEAR) > NE(i) + NH(i)) || ( rand > DNSn )) % nest with nestlings fails

cDOA(i) = DAY;

DOA(i,cNUM) = cDOA(i);

FLE(i,cNUM) = 0; % nestlings die, fledge 0

break

end

DAY = DAY + 1;

end

end

% -------------- Post-fledging Survival

IND(i,cNUM) = FLE(i,cNUM);

if ( cDOA(i) == 0 )

suNests(i)=suNests(i)+1;

nrIND = FLE(i,cNUM);

FLD(i,cNUM) = DAY;

for j=1:nrIND

if ( WL(DAY,YEAR) > NE(i) )

if (rand > PFSwet)

IND(i,cNUM) = IND(i,cNUM) -1;

end

else

if (rand > PFSdry)

IND(i,cNUM) = IND(i,cNUM) -1;

end

end

end % for j=1:nrIND

NSTLt(i) = NSTLt(i) + NSTL(i,cNUM); % total number of nestlings for female i

FLEt(i) = FLEt(i) + FLE(i,cNUM); % total number of fledglings for female i

INDt(i) = INDt(i) + IND(i,cNUM); % total number of ind young for female i

end % if cDOA(i) == 0 )

end % function renest(i,DFEx)
